# A biogeographic comparison of two convergent bird families

**DOI:** 10.1371/journal.pone.0335195

**Published:** 2025-10-24

**Authors:** Abdel H. Halloway, Christopher J. Whelan, Çağan H. Şekercioğlu, Joel S. Brown

**Affiliations:** 1 Department of Biological Sciences, University of Illinois at Chicago, Chicago, Illinois, United States of America; 2 Department of Metabolism and Cancer Physiology, Moffitt Cancer Center, Tampa, Florida, United States of America; 3 Department of Biology, University of Utah, Salt Lake City, Utah, United States of America; 4 Department of Molecular Biology and Genetics, Koç University, Istanbul, Türkiye; 5 KuzeyDoğa Derneği, Istasyon Mahallesi, Ismail Aytemiz Caddesi, Kars, Türkiye,; 6 Department of Integrated Mathematical Oncology, Moffitt Cancer Center, Tampa, Florida, United States of America; Universidad de Chile, CHILE

## Abstract

Convergence between species and entire clades can occur due to shared environmental conditions and shared resource use. Comparisons of biogeography between convergent clades and taxa may reveal some of these properties unique to each taxon. We sought to characterize and compare the global scale biogeography of hummingbirds (family Trochilidae), which possess unique adaptations for nectar feeding, with sunbirds (family Nectariniidae), which also feed on nectar but are more generalist in their feeding ecology. We collected the latitudinal and elevational range of all species in both clades to create species distributions along those gradients by way of empirical cumulative distribution functions. We compared those distributions to see 1) if they differed, by way of minimum difference estimation and 2) how they differed, by way of non-linear regression. Hummingbirds are shown to extend into higher elevations and latitudes compared to sunbirds, and better maintain their species number in these more extreme environments. We provide possible reasons for these patterns including dispersal limitation, land area, diversity of resources, and climatic conditions. In one particularly interesting hypothesis, we propose that hummingbirds’ unique adaptations for nectar feeding allow them to exploit resources more efficiently, gain higher intrinsic fitness, and therefore speciate and spread into more extreme climates than less efficient nectar feeding sunbirds.

## Introduction

Convergent evolution provides startling examples of how natural selection shapes species’ traits to optimize fitness as species which have different evolutionary histories come to share morphologies, physiologies, and behaviors due to shared ecologies [1–4]. Typically, evolutionary convergence occurs due to coexistence in similar environmental conditions and habitats. Such convergent species have similar adaptations to deal with and maintain fitness in comparable environmental conditions, e.g., the sidewinding snakes *Bitis peringueyi* of southern Africa, *Cerastes cerastes* of North Africa and the Middle East, and *Crotalus cerastes* of the deserts of the southwest United States and Mexico all use a sidewinding motion to travel across loose desert sand [5,6]. Evolutionary convergence can also occur due to similarity in resource exploitation. Species which share the same resources or exploit resources with similar features and characteristics often have similar adaptations to exploit those resources. This latter form of convergence differs in two key ways. First, if higher taxa (e.g., families and orders) share similar resource features, then convergence can occur at these higher taxa beyond the species level. Second, if the resource is not tied to environmental conditions or can exist in a broad range of environmental conditions, then convergent taxa, especially higher order taxa, may share biogeographic distributions independent of the environmental conditions. In this latter case, the environmental conditions and habitats shared by convergent taxa do not arise through extrinsic factors imposed on the species but are more intrinsically driven. Altogether, this suggests that biogeographic comparisons may be able to shine a light on the unique natural histories of convergent taxa.

One striking example of convergent taxa is that of nectarivorous birds. These include the hummingbirds (order Apodiformes, family Trochilidae) found in the Americas and the passerine nectarivores including the Hawaiian honeycreepers (order Passeriformes, family Fringillidae, subfamily Carduelinae), Australian honeyeaters (order Passeriformes, family Meliphagidae), and the Asian and African sunbirds (order Passeriformes, family Nectariniidae) along with a few other smaller clades like the non-Hawaiian honeycreepers (order Passeriformes, family Fringillidae, genus *Cyanerpes*). Some or all members of these families show convergent adaptations for nectarivory, particularly elongated bills and extensile tongues, but do differ in how specialized they are in foraging adaptations and ecology. These differences in specialization may present themselves as one or more of the families being found in a greater variety of environmental conditions and a wider geographic range.

Of particular interest are the hummingbirds and sunbirds. They are among the most speciose of the clades and display the greatest biogeographical ranges. Between the two, hummingbirds display a stronger mutualistic co-adaptation with flowers [7,8]. All hummingbirds feed almost exclusively on nectar, only supplementing protein intake by eating small insects [9]. As such, they have evolved distinct anatomical and morphological features suited to nectar foraging. In addition to an elongated bill and extensile tongue, the hummingbird’s tongue acts as a micro-pump for reaching and gathering nectar [10,11]. They possess large breast muscles (25–30% of body weight) particularly the supracoracoideus which generates power for the upstroke [12,13], skeletal architecture common to Apodiformes, and dense erythrocyte counts for delivering a steady supply of oxygen to feed extremely active skeletal and heart muscles [7,12,13]. Specialized wings allow hummingbirds to hover and fly backwards.

Sunbirds, on the other hand, are not as tightly adapted to nectar feeding with many species supplementing their diet with insects, seeds, fruit and flower heads, and others being largely insectivorous [14]. They also show large variation in bill and flight morphology with the flowerpeckers and the *Hedydipna* and *Hypogramma* sunbirds having broad, flat tongues. In addition, all sunbirds lack the musculoskeletal architecture to hover efficiently like hummingbirds, and many must perch to feed [8]. Furthermore, sunbirds use intralingual suction as their means to gather nectar versus the hummingbird micro-pump [15–17]. As both families are non-sympatric and have independently diversified, this makes them ideal convergent clades for biogeographic comparison.

In this study, we compare the biogeography of hummingbirds and sunbirds by analyzing each family’s latitudinal and elevational distribution and reveal similarities and differences in geographic distribution between these two convergent families. By doing so, we hope to reveal whether the differences in foraging ecology between hummingbirds and sunbirds affect their distribution.

## Materials and methods

To compare the biogeography of hummingbirds and sunbirds, we gathered the latitudinal and elevational range of all species from each family. These gradients generally show species number declining towards higher altitudes and more extreme latitudes [18–22]. Elevational ranges came from a global bird ecology database covering all the bird species of the world [23–26] while latitudinal ranges of species’ current ranges were taken from shapefiles downloaded from BirdLife International and NatureServe with data extracted using R packages “sp”, “raster”, “rasterVis”, “maptools”, and “rgeos” [27]. All latitudinal extremes located in the Southern hemisphere were converted to negative values, and latitudinal maxima and minima were rounded up and down to the nearest integer respectively. For example, the hummingbird species *Amazilia amabilis* which ranges from 14.17N to 3.98S would have its range taken as 15 to −4. An additional measure of distance from the equator, hereafter referred to as “polewardness”, was created. If a species’ range crossed the equator, then the poleward range was taken to be from 0 to the maximum distance from the equator. For *A. amabilis*, its poleward range would be 0–15 degrees. The poleward range of a solely Northern or Southern hemispheric species would simply be the absolute value of its latitudinal range.

With ranges in hand, we compared the families in two ways. First, we compared several empirical cumulative distribution functions (ECDFs) based upon the three geographical properties (elevation, latitude, and polewardness) for each family. Each ECDF started from sea level, the South Pole, and the equator and traced to higher altitudes, northward, and more extreme poles. For each geographical property, three ECDFs were created with a species’ presence based on the minimum, the midpoint, and the maximum of its range. Since species which cross the equator are not necessarily symmetric around it, the midpoint of a species poleward range may not accurately reflect its bias towards the equator or poles. Therefore, we created another measurement of species presence for polewardness, its expected value (see SI). This led to ten different ECDFs for each family: minimum, maximum, and midpoint for elevation, latitude and polewardness, and the additional measure of expected polewardness. Each type of ECDF was then compared between families using the Kolmogorov-Smirnov and Anderson-Darling minimum difference estimation (MDE) tests with the assumption that the hummingbird ECDF is less than the sunbird ECDF (one-tailed tests).

The ECDF analysis tells us whether the distributions differ, not necessarily how they differ. Therefore, we additionally sought to characterize each family’s distribution by measuring changes in species number with polewardness and elevation. To do so, we first counted the number of species in poleward and elevation intervals of 5 degrees and 500 meters for each family. If the edge of a species’ range was at the cutoff point of the interval, it would be considered present in the lower interval but not in the upper interval due to previous rounding. In the example with *A. amabilis*, this means that the species is counted in the 10–15 degree interval but not in the 15–20 degree interval. The frequency data were then normalized such that the interval with the highest number of species became 1 to remove any effect of total species number. This gave us four sets of data based on a 2x2 factorial: sunbird and hummingbird polewardness and elevation. A logistic curve (eq. 1) was then fitted onto each of the four sets of data – the normalized species number, $S_{N}$, per interval vs. the midpoint of each interval – with variables a  and b determining position and steepness of the curve respectively:


$$S_{N}=\frac{\text{1}}{\text{1}+ae^{bx}}$$ (1)

We then found the inflection point and the two points of the maximum magnitude of curvature (MMC points) for each curve. Inflection points indicate how well each family maintains species number while MMC points give us the start and end of the decline in species number. The functions and their key points characterize the shape of each family’s gradient.

## Results

**Fig 1:**
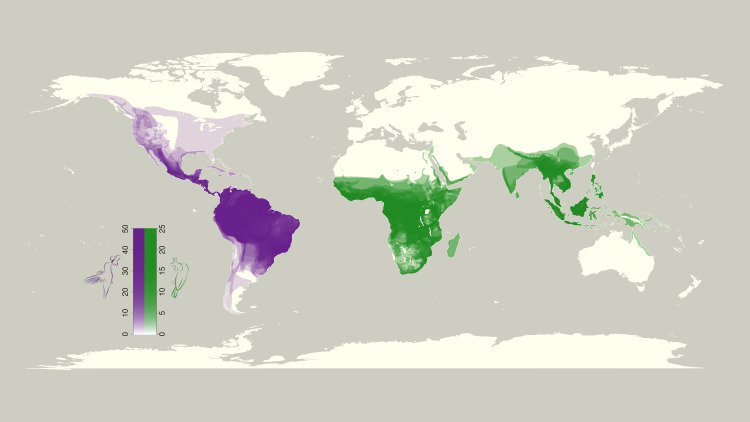
Geographic Map of Family Ranges. A map of species number of hummingbirds (purple) and sunbirds (green), normalized to the maximum species number per area of each clade. Richer colors represent greater species number. Scales are chosen to reflect the difference in overall species number of each taxon. Hummingbirds not only have higher species number but also extend farther latitudinally. This figure was created with the ‘map’ function from the ‘maps’ package in *R* and the shapefiles from BirdLife International and NatureServe [27].

**Fig 2:**
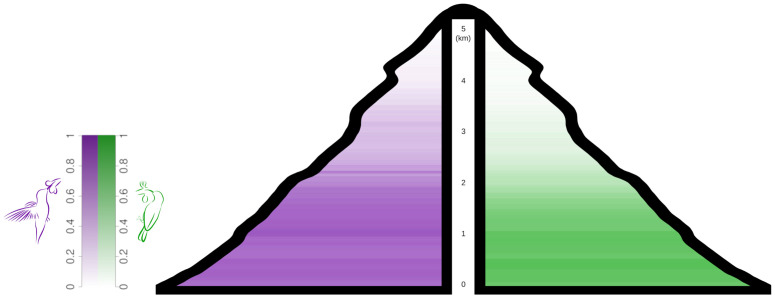
Figure of Elevational Gradients for the Families. Changes in relative species number with elevation (normalized to each clade’s maximum number at a given elevation) for hummingbirds (purple) and sunbirds (green). Though both clades extend to similar altitudes, hummingbirds maintain species number at higher elevations as denoted by the richer color.

**Fig 3:**
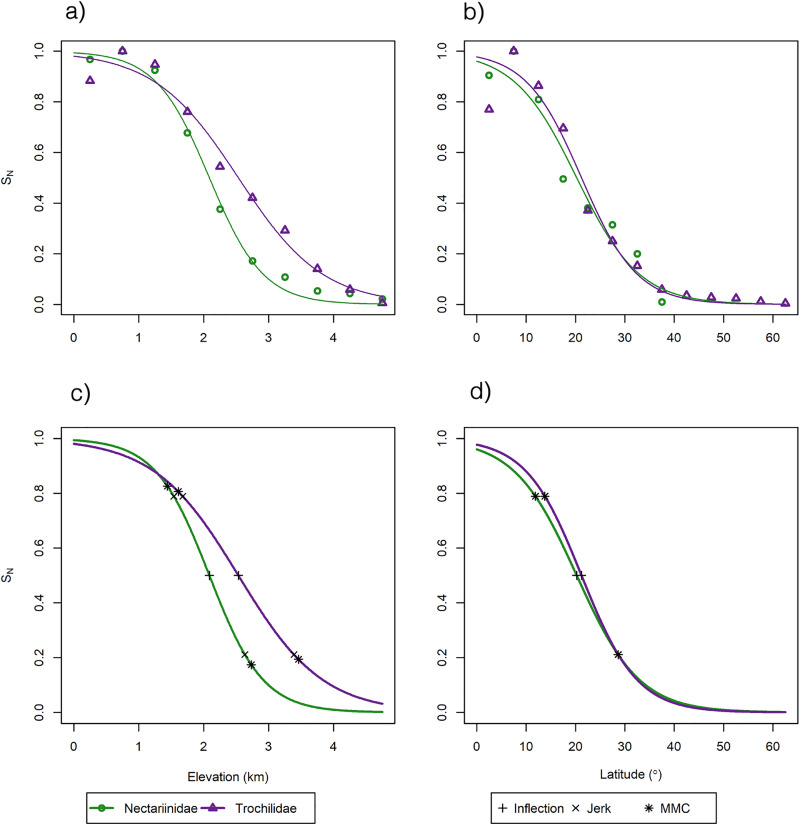
Normalized Species Richness of the Families. A plot of the normalized species number $S_{N}$ of hummingbirds (Trochilidae) and sunbirds (Nectariinidae), along with the fitted line, for elevation (a, c) and “polewardness” (b, d). Triangles and purple lines denote hummingbirds, and circles and green lines denote sunbirds. Hummingbirds maintain species number at higher elevations and mid-latitudinal ranges, and extend farther latitudinally than sunbirds. Inflection (cross) and MMC points (asterisks) also are shown (c, d). Inflection points come later in hummingbirds than sunbirds. With regard to elevation, hummingbird $S_{N}$ and sunbird $S_{N}$ start their decline at a similar elevation but hummingbird $S_{N}$ declines more slowly. With latitude, sunbird $S_{N}$ declines earlier than hummingbird $S_{N}$.

**Fig 4:**
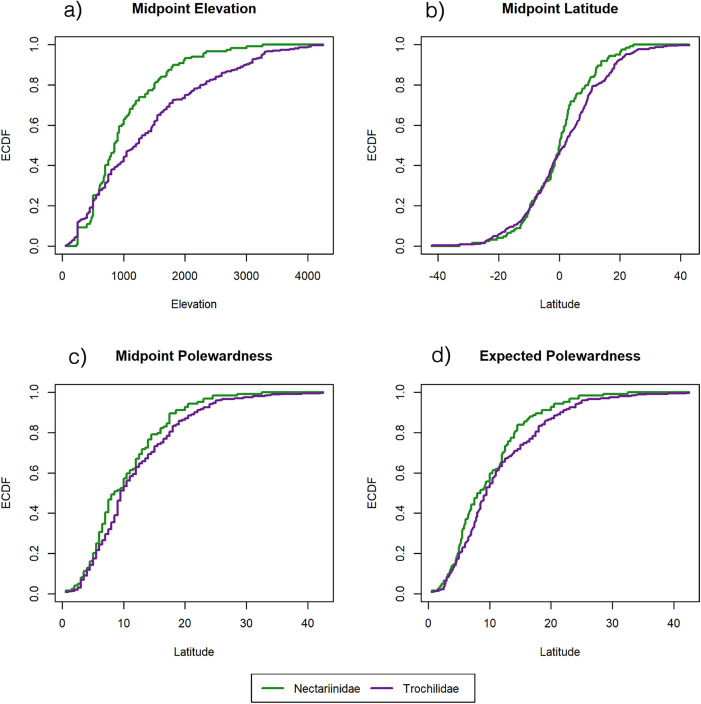
ECDF Figures. A plot of four select ECDFs used to compare hummingbird (Trochilidae) and sunbird (Nectariinidae) distributions. Purple lines indicate hummingbirds and green lines indicate sunbirds. Hummingbird ECDFs almost entirely lie below sunbird ECDFs, with the greatest difference occurring with respect to elevation measures.

Broadly, our results show that hummingbirds extend farther poleward and higher in elevation than do sunbirds. Hummingbirds extend from 62 degrees north to 56 degrees south and up to 5000 m in elevation (S1 and S2 Tables in S1 File; Figs 1 and 2). Sunbirds, on the other hand, extend only from 36 degrees north to 40 degrees south and up to 4880 m in elevation (S1 and S2 Tables in S1 File; Figs 1 and 2). Both families show the same general pattern of an initial increase in species number followed by a decline moving poleward and to higher altitudes (Fig 3a and 3b). In addition, hummingbirds maintain their species number at higher elevations and more extreme latitudes than sunbirds. ECDF results confirm this difference in biogeography between hummingbirds and sunbirds with elevation constituting the greatest difference (S3 Table in S1 File, Fig 4).

Both hummingbirds and sunbirds reach approximately the same maximum elevation, around 5000m (S1 Table in S1 File; Fig 4a). Even though both hummingbirds and sunbirds extend to roughly the same elevation, hummingbirds have a higher normalized species number at higher elevations compared to sunbirds. The inflection point for sunbirds occurs at 2087m and hummingbirds at 2533m (S4 Table in S1 File; Fig 3c). Sunbirds and hummingbird species number values both start to decline around the same elevation –1764 and 1898m respectively – but sunbirds plateau at a lower elevation compared to hummingbirds –2410m vs. 3458m respectively. This indicates a more gradual decline in the normalized species number of hummingbirds (S4 Table in S1 File; Fig 3c).

Regarding latitude, hummingbirds occur farther from the equator than do sunbirds, 60–65 degrees vs. 35–40 degrees respectively (S2 Table in S1 File; Fig 4b). Also, hummingbird normalized species number is at its greatest divergence from sunbird normalized species number at mid-latitudinal ranges. The hummingbirds’ inflection point is 22.14 degrees latitude versus 18.92 degrees for sunbirds (S4 Table in S1 File; Fig 3d). Hummingbird diversity begins to decline further from the equator than do sunbirds –14.99 and 9.44 degrees respectively. Both plateau around the same latitude – 29.29 vs. 28.39 degrees respectively (S4 Table in S1 File; Fig 3d).

## Discussion

We characterized and compared the geographical distributions of two convergent nectarivorous bird families: sunbirds (Nectariniidae) and hummingbirds (Trochilidae). Our results show that hummingbirds show a wider biogeographic distribution compared to sunbirds. As one moves higher in elevation and towards the poles, hummingbirds maintain their species number more than sunbirds. Though extending to roughly the same elevational maximum, normalized hummingbird species number declines at a much slower rate than that of sunbirds. The same is true for latitude with hummingbirds extending into more extreme latitudes (farther north and south) than sunbirds. Despite their convergence, hummingbirds and sunbirds do have differences in foraging ecology with hummingbirds being more specialized in nectar foraging. The difference in biogeography suggests that the stronger and more efficient adaptations of hummingbirds outweigh the more generalist feeding niches of sunbirds to create a more extensive geographic distribution.

Adaptations are known to affect the range size and distributions of species [28–31]. Differences in adaptations can open new ecological opportunities, changing the fundamental niche of the clades [32]. Adaptations which do so are known as key adaptations. Key adaptations can lead to a divergence in fundamental niches, e.g., the ankle bones of the grandorder Euarchonta (four orders of mammals including primates) that promote arboreal living. Key adaptations may also allow a taxon to expand beyond its original range of environments and have a greater fundamental niche by increasing the fitness of a clade at the margins of its range [33,34]. Examples include the retractable necks among turtles of the suborder Cryptodira, which protect them from predation, and the infrared-sensing pits among vipers of the subfamily Crotalinae, which allows them to “see” mammals at night. This greater environmental range may manifest as a greater geographical distribution. Looking at the convergent mice genera *Peromyscus* and *Apodemus*, *Peromyscus* inhabits colder, more arid, and higher elevation habitats as compared to *Apodemus. Peromyscus* has a more efficient and widely used torpor state (Montgomery 1989). Another effect of key adaptations is that they may drive the clade’s radiation, making it more speciose [32]. In a species rich clade, some species may occur in more extreme environments based on chance alone. Overall, we suspect that the specialized nectar feeding of hummingbirds represents an evolutionary breakthrough that increases their intrinsic fitness compared to sunbirds. While generalized species may be able to take advantage of a greater diversity of resources, if resource turnover between habitats is low, comparative fitness between habitats remains similar for both clades, at which point absolute fitness within a habitat driven by the ability to capture and assimilate resources matters more.

We can speculate on what adaptations may constitute a key adaptation that allows hummingbirds to exploit a wider range of environments. Though hummingbirds and highly specialized sunbirds show many similarities, they do differ in specific aspects of physiology, morphology, and anatomy. Likely, the key adaptation deals with the differences in their foraging, specifically how they feed and how they fly. With feeding, one possibility for hummingbirds’ key adaptation may be their unique tongues. The tongues of hummingbirds have recently been shown to act as micropumps, a way of quickly and efficiently gathering nectar from flowers, in contrast to the previously assumed capillary action [10,11]. This unusual feeding method may allow hummingbirds to more efficiently gather nectar compared to sunbirds. While sunbird feeding has not been studied as extensively as hummingbird feeding, studies indicate that hummingbirds and sunbirds gather nectar at seemingly comparable rates, suggesting that the amount gathered is not the key difference (Hainsworth [35], 1973; Schlamowitz et al. [36], 1976; Paton and Collins [37], 1989; Guevara et al., 2015). If the tongue is the key adaptation, it will be for the fact that micropumping requires no energy expenditure on the part of hummingbirds, which removes a cost, while sunbirds intake nectar through suction, a potentially energetically expensive system [15,38,16].

Another possible key adaptation separating hummingbirds from sunbirds is the hummingbird’s ability to hover and fly in all directions [7]. Adaptations for hovering include shortened arm bones, longer hand bones, a relatively fixed V-shaped arm position, a shallow ball-and-cup joint between the coracoid and sternum, a large sternum with a deep keel onto which large breast muscles – pectoralis and particularly the supracoracoideus which is much larger relative to the pectoralis than most birds – attach, and red blood cells and hemoglobin adapted for higher-oxygen affinity and carrying capability [12,13,39–42]. All these anatomical features are adaptations to stiff-winged flight are seen to a lesser extreme within other bird families of the order Apodiformes [39–41].

What truly differentiates the flight of hummingbirds is the axial rotation of the humerus and wrist bones during flight [39]. Hummingbirds can create lift on the upstroke – in addition to the downstroke seen in all birds – due to wing inversion caused by axial rotation of the wrist [42]. Wrist flexibility comes from changes in carpal structure and the deletion of key ligaments and is seen in birds outside of Apodiformes [39,43]. Additional power for each downstroke and upstroke also comes from the axial rotation of humerus, driven by the pectoralis, supracoracoideus, and other muscles [39,42,44]. The humerus can rotate up to 180º due to a unique humeroscapular joint [40,45]. In hummingbirds, the humeral connection with the shoulder joint is at the extended capital tuberculum instead of the humeral head, a feature unique to them (Karhu 1999, Videler 2006). Together, this suite of adaptations allows hummingbirds to hover with agility when foraging [46].

Other adaptations may also benefit hummingbirds in secondary ways. For example, hummingbirds sustain flight more efficiently at higher altitudes, likely due to their denser erythrocyte count, expanding their fundamental niche to higher elevations [47]. We feel though that hovering remains the likeliest candidate for the hummingbird’s key adaptation. Many of the musculoskeletal changes are seen only in Apodiformes with a few features like the modifications to the humeral head seen only in Trochilidae. Such efficient hovering is likely evolutionarily implastic and a derived trait of Trochilidae that arose only once among Aves. Through this adaptation, hummingbirds have fundamentally changed the rules of their nectarivory. They exist as a new bauplan while sunbirds are still effectively a derived passerine [34,48].

There could be many reasons why hummingbirds developed their key adaptation. Hummingbirds underwent an expansive radiation during the uplift of the Andes beginning around 10 mya [49]. Living in such rapidly changing conditions could have necessitated the evolution of a more efficient foraging system. As mentioned earlier, greater oxygen capacity is beneficial to both hovering and living in low oxygen conditions. There is also the possibility that the rise of the Andes freed up niche space that would have otherwise been taken up by a competing family like hawkmoths (Sphingidae), a sort of ecological constraint [50]. These factors, along with hummingbirds’ evolutionary history, may combine to lead to the evolution of hovering [46]. Furthermore, sunbirds may face their own internal constraints, genetic or otherwise, preventing them from evolving the hummingbird’s key adaptations [51]. Whatever the case may be, our results suggest the evolution of hovering (or perhaps the integrated combination of multiple adaptations) allowed hummingbirds to more efficiently take advantage of a resource and expand their fundamental niche.

While we believe that the adaptations intrinsic to each clade have a significant role in determining their biogeography, we also recognize that other, external factors may play a role. These include but are not limited to land area, number and diversity of resources, climatic conditions, and dispersal barriers. While we have not tested for these influences, we can reject some of these hypotheses based upon our analyses. First, with regard to land area, South America extends further south than Africa and Australia which does allow hummingbirds to extend further south than sunbirds. However, Europe and Asia extend just as far north as North America and yet still sunbirds are unable to extend as far north as hummingbirds. One reason may be mountains, which primarily run along the north-south axis in the Americas as opposed to east-west in Europe and Asia. Furthermore, the Mediterranean Sea, and extreme deserts like the Sahara and Arabian deserts may act as dispersal barriers for sunbirds. However, we note that hummingbirds are frequently found in montane and desert habitats where they maintain higher relative species numbers at higher elevations than do sunbirds. Interestingly, hummingbirds have higher species number in the mountains of western North and South America compared to the flat-lying eastern regions. Hummingbirds also show high species numbers in desert areas such as the Sonoran and Chihuahuan deserts. This is also true of abundance, in that hummingbirds show high combined population densities at higher altitudes in the Southwestern United States [52]. Furthermore, while these explanations may influence the differences in latitudinal gradients, they cannot explain the differences in elevational gradients. They also mostly represent proximate and not ultimate factors as they cannot explain why one clade is better able to deal with environmental conditions than the other.

We also acknowledge the role data collection may play in the analysis. The Americas and Western countries are more sampled in a variety of taxa than in Africa, Asia and parts of South America. Therefore, with range expansions, more recent and updated data may expand the latitudinal and elevation ranges of sunbirds more than hummingbirds. While this may affect our analysis on elevation, we believe it is highly unlikely to affect latitudinal ranges since highly sampled Europe and Australia still see few to no sunbirds.

The real test of adaptations affecting biogeography would come from experiments that directly compare growth and reproduction under the same conditions. This would happen when species from the two clades become sympatric. As such, species invasions may offer such a test. For example, European Lumbricid earthworms have colonized parts of North America that are farther north than their American counterparts [53]. Both sets of earthworms are ecological equivalents and have convergent features to fill the role of soil turners. The invasive European earthworms, though, are known to tolerate environmental stress through protective cocoons during times of drought, and the high glucose and glycogen contents in their cells prevent freezing during winter [54,55]. These adaptations may have allowed European earthworms to colonize the colder climes of Canada and expand their range beyond the North American species, an expansion that is still ongoing [56].

Through biogeographic analysis, we show that hummingbirds have a larger geographical distribution and inhabit more hostile climes than sunbirds. We hypothesize that this is due to the presence of a key adaptation in hummingbirds, like hovering, which allows them to have a more specialized yet efficient foraging ecology, leading to a higher intrinsic fitness compared to sunbirds. While not definitive, our analysis provides evidence for and supports our hypothesis. Further and more detailed comparisons of the evolutionary ecology of hummingbirds and sunbirds will help refine the causes of their biogeographic differences. Going forward, biogeographic comparison may reveal itself to be a powerful tool to reveal differences between clades and shed light on the interaction between adaptation and environment.

## Supporting information

S1 File Detailed Table of Results and Methodology. This file contains four tables of the detailed results for the elevational and latitudinal comparisons between sunbirds (Nectariniidae) and hummingbirds (Trochilidae) plus a more detailed description of the methodology.
(PDF)
