# Supplementary material for: A biogeographic comparison of two convergent bird families
Source: PLoS One. 2025 Oct 24;20(10):e0335195. doi: 10.1371/journal.pone.0335195 (PMC12551894; doi:10.1371/journal.pone.0335195)
Supplement: S1 File — This file contains four tables of the detailed results for the elevational and latitudinal comparisons between sunbirds (Nectariniidae) and hummingbirds (Trochilidae) plus a more detailed description of the methodology. (PDF) [file pone.0335195.s001.pdf]

SI Table 1: Normalized species richness per elevation interval of sunbirds and hummingbirds

Elevation (meters)	Sunbirds	Hummingbirds
[0 , 500]	0.967741935	0.883040936
(500 , 1000]	1	1
(1000 , 1500]	0.924731183	0.947368421
(1500 , 2000]	0.677419355	0.760233918
(2000 , 2500]	0.376344086	0.543859649
(2500 , 3000]	0.172043011	0.421052632
(3000 , 3500]	0.107526882	0.292397661
(3500 , 4000]	0.053763441	0.140350877
(4000 , 4500]	0.043010753	0.058479532
(4500 , 5000]	0.021505376	0.005847953

SI Table 2: Normalized species richness per latitudinal interval of sunbirds and hummingbirds

**Latitude (degrees)**	**Sunbirds**	**Hummingbirds**
[0 , 5]	0.904761905	0.76953125
(5 , 10]	1	1
(10 , 15]	0.80952381	0.86328125
(15 , 20]	0.495238095	0.6953125
(20 , 25]	0.380952381	0.37109375
(25 , 30]	0.314285714	0.25
(30 , 35]	0.2	0.15234375
(35 , 40]	0.00952381	0.05859375
(40 , 45]	0	0.03515625
(45 , 50]	0	0.02734375
(50 , 55]	0	0.0234375
(55 , 60]	0	0.01171875
(60 , 65]	0	0.00390625

SI Table 3: The results of the ECDF comparisons (MDE tests) between families. *Italics* indicate the centrality based ECDFs. **KS** stands for Kolmogorov-Smirnov and **AD** stands for Anderson-Darling. NS indicates that the result was not significant.

**ECDF Type**	**Hbird Num**	**Sbird Num**	**KS D-statistic**	**KS Significance**	**Standardized AD Criterion**	**AD Significance**
Minimum Elevation	309	119	0.331783199	p<0.001	31.482	p<0.001
Maximum Elevation	309	119	0.163280846	p<0.05	3.2666	p<0.01
Midpoint Elevation	309	119	0.214598461	p<0.001	8.685	p<0.001
Minimum Latitude	365	124	0.234423332	p<0.001	10.207	p<0.001
Maximum Latitude	365	124	0.069443217	NS	0.49404	NS
Midpoint Latitude	365	124	0.156407424	p<0.05	1.7652	p<0.05
Minimum Polewardness	365	124	0.242222713	p<0.001	14.455	p<0.001
Maximum Polewardness	365	124	0.073486522	NS	0.71488	p<0.1
Midpoint Polewardness	365	124	0.14719399	p<0.05	1.4571	p<0.05
Expected Polewardness	365	124	0.13121962	p<0.05	1.8851	p<0.05

SI Table 4: The values of a and b, the inflection point, and the MMC points for each of the models along with their significance. RSS is the residual sum of squares for each model, and RSE is the residual standard error. It should be noted that all residuals fall between 0 and 1 which may exaggerate the RSS.

**Model**	**a**	**b**	**Inflection**	**MMC #1**	**MMC #2**	**RSS**	**RSE**
NectarElev	0.00655[d]	2.40963[a]	2.087005	1.441369	2.73264	0.010254	0.035802
TrochElev	0.02024[c]	1.53943[a]	2.533536	1.608667	3.458399	0.022979	0.053594
NectarLat	0.04133[c]	0.15735[a]	20.24934	11.872	28.62667	0.04463	0.063696
TrochLat	0.02318	0.17739[a]	21.22319	13.79037	28.656	0.054938	0.070671

a: $p<0.001$, b: $p<0.01$, c: $p<0.05$, d: $p<0.1$

Additional Methods

More on Latitudinal and Elevational Gradients

Besides the geographic and environmental reasons that make latitudinal and elevational gradients useful for this study, there are other methodological features that make them particularly useful for this analysis. Spatial comparisons over a wide geographic range are better able to indicate the presence of key adaptations, as they cover many environmental variables – reducing the chance of a false negative – but are not inherently correlated to the adaptation in question – preventing false positives. Additionally, elevational comparisons offer a stronger comparison between families. This is because a random distribution of the range sizes and locations within a bounded geographical space of a group of species will generate a hump-shaped gradient broadly resembling (though not identical to) latitudinal gradients (1). Elevational gradients occur in bounded geographical space but present a skewed distribution indicating the lack of random processes. Therefore, any difference between the families is likely an effect of their natural histories.

Expected Polewardness

The measure of expected polewardness was calculated with the assumption that abundance was stacked for species whose ranges crossed the equator. We assumed a uniform distribution of abundance across each species range. We calculated the expected value of this stacked uniform distribution seen in eq. (1) where min and max stand for the absolute value of minimum and maximum latitude respectively before folding.

$$\frac{1}{2} * \frac{min^2 + max^2}{(min + max)} \tag{1}$$

1. Willig MR, Lyons SK. An analytical model of latitudinal gradients of species richness with an empirical test for marsupials and bats in the New World. Oikos. 1998 Feb 1:93-8.
